# Role of Exclusive Enteral Nutrition in the Preoperative Optimization of Patients With Crohn's Disease Following Immunosuppressive Therapy

**DOI:** 10.1097/MD.0000000000000478

**Published:** 2015-02-06

**Authors:** Yi Li, Lugen Zuo, Weiming Zhu, Jianfeng Gong, Wei Zhang, Lili Gu, Zhen Guo, Lei Cao, Ning Li, Jieshou Li

**Affiliations:** From the Department of General Surgery, Jinling Hospital, Medical School of Nanjing University, No. 305 East Zhongshan Road, Nanjing, PR China (YL, LZ, WZ, JG, WZ, LG, ZG, LC, NL, JL).

## Abstract

We conducted a study to evaluate the impact of the exclusive enteral nutrition (EEN) on perioperative outcome in Crohn's disease (CD) patients following immunosuppressive therapy.

Patients with CD followed at a referral center between January 2001 and March 2014 who underwent abdominal surgery were identified. Patients were divided into 4 groups: patients not exposed to immunosuppressive agents in the previous 8 weeks before surgery (group 1); patients received immunosuppressive medications without preoperative drug-free interval (group 2); patients had preoperative immunosuppressants-free interval (group 3); patients treated with adding EEN to preoperative immunosuppressants-free interval regimen (group 4). Urgent operation requirement, stoma creation, postoperative complications, readmission, and reoperation were compared in patients among groups.

Overall, 708 abdominal surgeries performed in 498 CD patients were identified. Three hundred seventy-six (53.11%) surgeries performed in those receiving preoperative immunosuppressive medications. Compared with other groups, group 2 had increased postoperative complications, more frequent urgent operation, and higher rate of stoma creation. Patients in group 4 were found to have better outcome including lower rate of stoma creation (*P* < 0.05), and decreased incidence of postoperative complications (*P* < 0.05) compared with group 2 and group 3. Additionally, decreased urgent operation requirement (*P* < 0.05) and extended preoperative drug-free interval (*P* < 0.001) were observed in the group 4 than those in the group 3.

Preoperative optimization of CD following immunosuppressive therapy by EEN prolongs the immunosuppressants-free interval, reduces the risk of urgent surgery and reoperation, and most importantly, decreases complications after abdominal surgery.

## INTRODUCTION

Crohn's disease (CD) is a relapsing, transmural inflammatory bowel disease (IBD) that can affect the entire gastrointestinal tract from the mouth to the anus.^1^ Development of complications including strictures, abscesses, or fistulas is one of the typical presentations of CD.^2^ Surgical resection is an almost inevitable event through the course of CD and the rate of surgical intervention increases with disease duration.^3^ The rate of surgery is 20%–40% during the first year of the disease, 30%–70% at 10 years after diagnosis, and 70%–90% after 15 years of diagnosis.^4–6^ Surgery in CD is frequently performed in those with factors associated with the increased risk of postoperative complications such as malnutrition status, presence of abdominal abscess, and preoperative immunomodulators use.^7–9^

Currently, the medical management for CD is directed suppressing the overactive immune response, and typically relies on combination therapy with immunosuppressive medications which can be generally categorized into 3 classes: steroids, immunomodulators, and biologics.^10^ Although studies evaluating the impact of preoperative immunosuppressive therapy on postoperative outcome in CD patients undergoing abdominal surgery have revealed conflicting results, immunosuppressive agents are potentially associated with the increased incidence of postoperative complications. Combination immunosuppressive therapy before surgery in patients with CD appears to be associated with an increase in postoperative morbidity.^11,12^ Some reports also found the increased incidences in both mortality and morbidity after biologic treatment.^13–15^

To minimize the potential influence of immunosuppressive therapy, withdrawal of immunosuppressive agents before surgical intervention to provide the drug window with the purpose of drug wash out is recommended in actual clinical practice. The importance of a preoperative drug-free interval which may reduce the risk of infections after surgery is suggested.^11^ However, because of the immunosuppressive agent withdrawal, the disease might exacerbate during the preoperative drug-free interval which may subsequently result in urgent surgical intervention. Urgent surgery is suspected to be associated with increased postoperative complications and the increased incidence of stoma creation.^16,17^ Therefore, medical alternatives for disease control during the immusupressants-free interval is required. Unfortunately, medications such as 5-ASA is less effective in patients receiving previous chronic immunomodulators although the association between 5-ASA and postoperative adverse outcome was rarely reported.

Exclusive enteral nutrition (EEN), which can provide 100% nutrients needs of a patient and is largely free from significant side effects.^18,19^ EEN therapy is shown to be beneficial and efficacious in inducing and maintaining remission for CD.^20^ In our institute, EEN is increasingly used in adult CD patients especially for preoperative optimization. For those with fistulizing disease, preoperative EEN therapy was associated with the reduced risk of intra-abdominal septic complications after operation in CD.^21^ For those with stricture disease, EEN therapy can effectively relieve inflammatory bowel stricture in CD.^22^ We recently found EEN could effectively induce the disease remission and improve health-related quality of life in adults with active CD which may be associated with its activity in abdominal fat modification.^23–25^

Preoperative optimization of CD patients following immunosuppressive treatment may decrease postoperative complications, and may avoid temporary stoma creation.^26^ However, there are few reports evaluating the effects of preoperative optimization on the perioperative outcomes in patients with CD. Furthermore, how to optimize these patients is an important and difficult issue for both gastroenterologist and colorectal surgeons, and which is still not well answered. In the routine clinical practice at our IBD center, patients are generally recommended to receive adding EEN to preoperative immunosuppressants-free interval regimen although some patients may refuse to use EEN because of personal choice or economic issue. According to our experience, patients receiving preoperative EEN regimen rarely develop postoperative morbidity. However, evidence of the efficacy and safety of the EEN regimen in the preoperative drug-free interval is largely unknown for CD patients undergoing abdominal surgery. The aim of this study was to evaluate whether preoperative optimization by EEN can result in a better outcome in immunosuppressants-treated CD patients.

## METHODS

### Patients

From January 2001 to March 2014, information of all patients with CD who underwent abdominal surgery in Department of General Surgery of Jinling Hospital were collected from a prospectively maintained IBD database. The Institutional Review Board of Jinling Hospital approved the project. All CD patients have a verified diagnosis according to conventional clinical, radiological, and endoscopic criteria, confirmed by the histological findings.^27,28^

Preoperative immunosuppressive medication use within 8 weeks before surgery was recorded and categorized into 3 classes: steroids, immunomodulators, and biologics. Patients were divided into 4 groups: patients not exposed to immunosuppressive agents in the previous 8 weeks before surgery (group 1); patients received immunosuppressive medications without preoperative drug-free interval (group 2); patients had preoperative immunosuppressants-free interval (group 3); patients treated with adding EEN to preoperative immunosuppressants-free interval regimen (group 4). Adding EEN to drug-free interval regimen was defined as EEN treatment from the time of immunosuppressive medication withdrawal to 1 day before surgical intervention. Patients were generally recommended to receive EEN treatment for 4 weeks. The EEN therapy was performed as previously described.^23,25^ During the period of EEN therapy, any other food and drink except water was forbidden. The enteral formula composing of maltodextrin, hydrolyzed whey protein peptide, fat, vitamins and trace elements was a commercially available Peptisorb Liquid (Nutricia, Amsterdam, the Netherlands). The enteral formula was infused continuously through a nasogastric tube. The daily calorie intake was 25–30 kcal/kg body weight and was gradually increased from one-third of amount to the full amount.

### Data Collection

Patients character such as age, disease duration, age at diagnose, disease location, disease behavior, current smoking status, preoperative medical therapy, CD-related surgical history, and body mass index (BMI) were collected. Operation-related information including indication for surgery, type of surgery (open or laparoscopic), stoma creation, number of anastomoses, emergent surgery, principle surgical procedure, operative time, and estimated blood loss were also collected. We were particularly interested in the duration of immunosuppressants-free interval, postoperative 30-day complications. Special interesting was also given to the reoperation and readmission.

### Outcome Measurement

Thirty-day postoperative complications were collected and divided into infectious complications (wound infection, intaabdominal abscess, anastomotic leak, fistula, urinary tract infection, pulmonary infection) and noninfectious complications (eg, ileus, bleeding, thrombolic events, dehydration, other). The primary outcome assessed was the infectious complication rate up to 30 days after surgery. Secondary outcome was the rate of noninfectious complications within 30 days postoperatively. We were also interested in the rate of stoma creation, urgent operation requirement, reoperation, and readmission.

### Statistical Analysis

All data were entered into a standardized database computer program and the statistical analysis was performed using SPSS 16.0 software (SPSS, Inc., Chicago, IL). A χ^2^ test or Fisher's exact test was used to compare the categorical variables. Continuous variables were expressed as the mean and standard deviation (SD) and were compared by using a Student's *t*-test test. One-way analysis of variance (ANOVA) was used for multiple group comparisons. Multivariate analysis using was used to identify the independent risk factors for postoperative complications. Independent variables for multivariate analysis included those with a significant influence in univariate analysis (*P* < 0.05). The odds ratio (OR) and 95% confidence interval were calculated for all the variables. All hypothesis testing was 2-sided with a *P* value of <0.05 was considered significant in all the analyses.

## RESULTS

### Patient's Character

**FIGURE 1:**
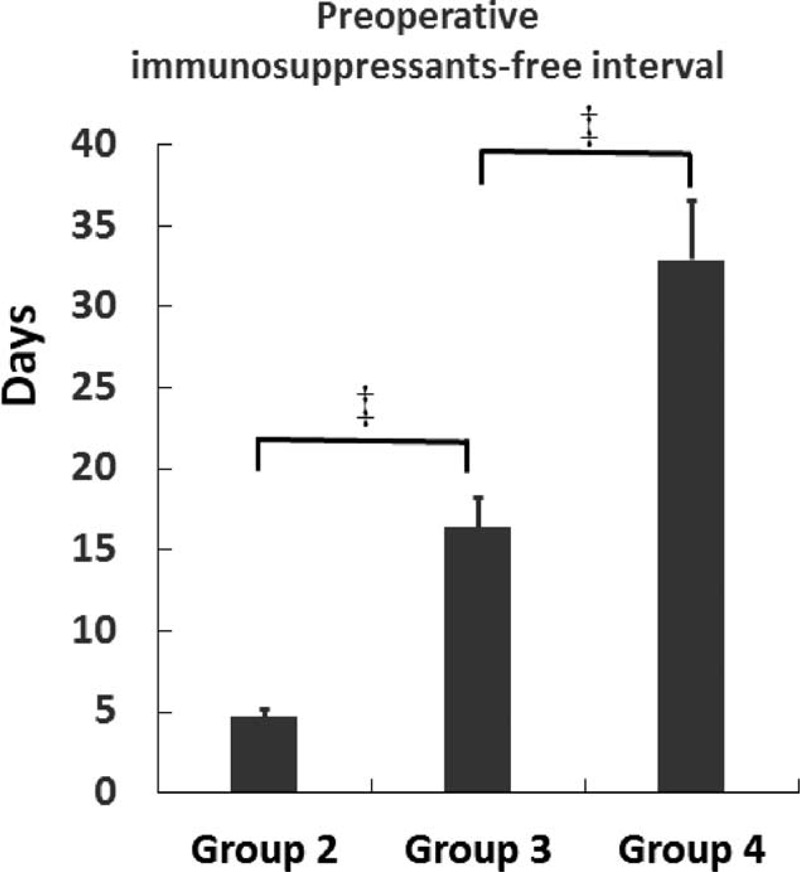
The preoperative immunosuppressants-free interval in group 2, group 3, and group4. ^‡^*P* < 0.001.

There were a total of 708 surgeries performed in 498 patients. Patients in group 1 were operated between 2001 and 2013, patients in group 2 received surgery from 2002 to 2012, patients in group 3 underwent surgical intervention between 2001 and 2004, and patients in group 4 were operated from 2001 to 2014. Three hundred seventy-six (53.11%) of these surgeries were performed among patients who received perioperative immunosuppressive agent. Clinical characteristics of the study population in each group are shown in Table 1. No significant differences were observed among patient groups in gender, age at surgery, disease duration, age at diagnosis, disease location, disease behavior, or previous CD-related surgery. In addition, there were no significant differences among group 2, group 3, and group 4 in terms of preoperative medical therapy or the number of immunosuppressive medications. However, patients in group 4 had increased BMI at the time of surgery when compared with those in group 1 (*P* = 0.003) and group 3 (*P* = 0.03). Furthermore, our data indicated patients in group 4 had longer preoperative drug-free interval (Figure 1).

**TABLE 1:**
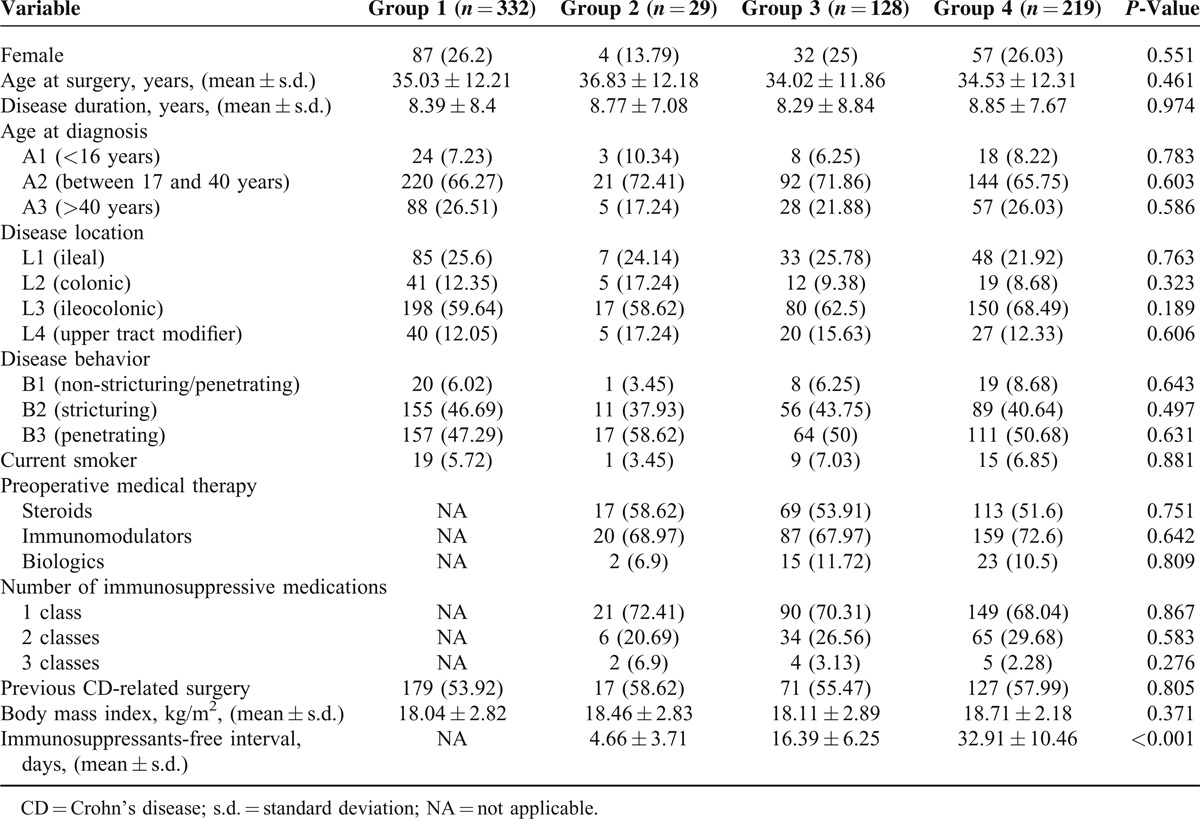
Clinical Features by Number of Classes of Immunosuppressive Medications

**FIGURE 2:**
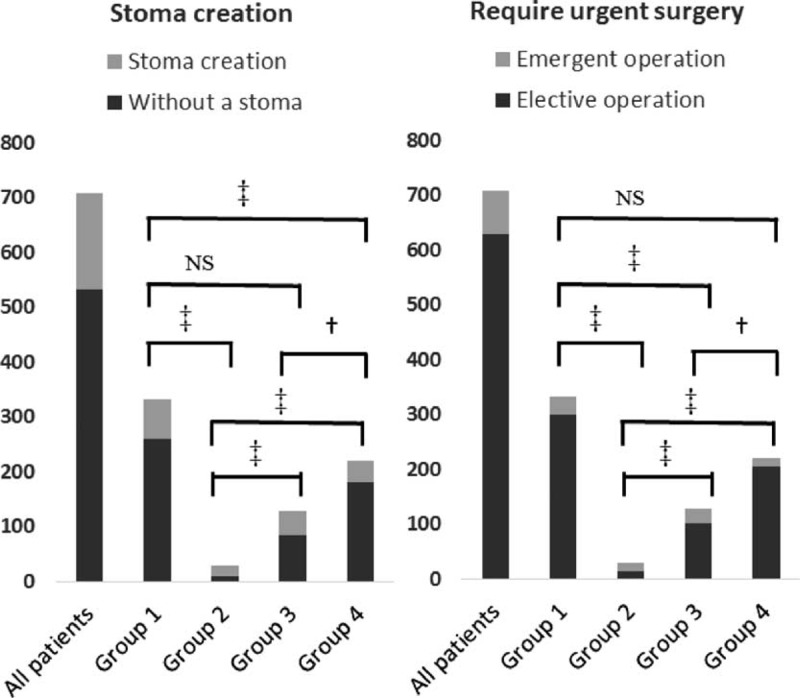
Stoma creation and urgent surgery requirement among groups. ^†^*P* < 0.05, ^‡^*P* < 0.001, and NS means not significant.

Operative details are presented in Table 2. There was no difference in surgical indication, type of surgery, number of anastomoses, principle surgical procedure, operative time, or estimated blood loss among groups. However, patients in group 2 were more likely to have fecal diversion, while patients in group 4 had a lower frequency of stoma creation (Figure 2). In addition, patients in group 2 had more requirement of emergent surgical intervention. However, decreased incidence of urgent operation was observed in group 4 (Figure 2).

**TABLE 2:**
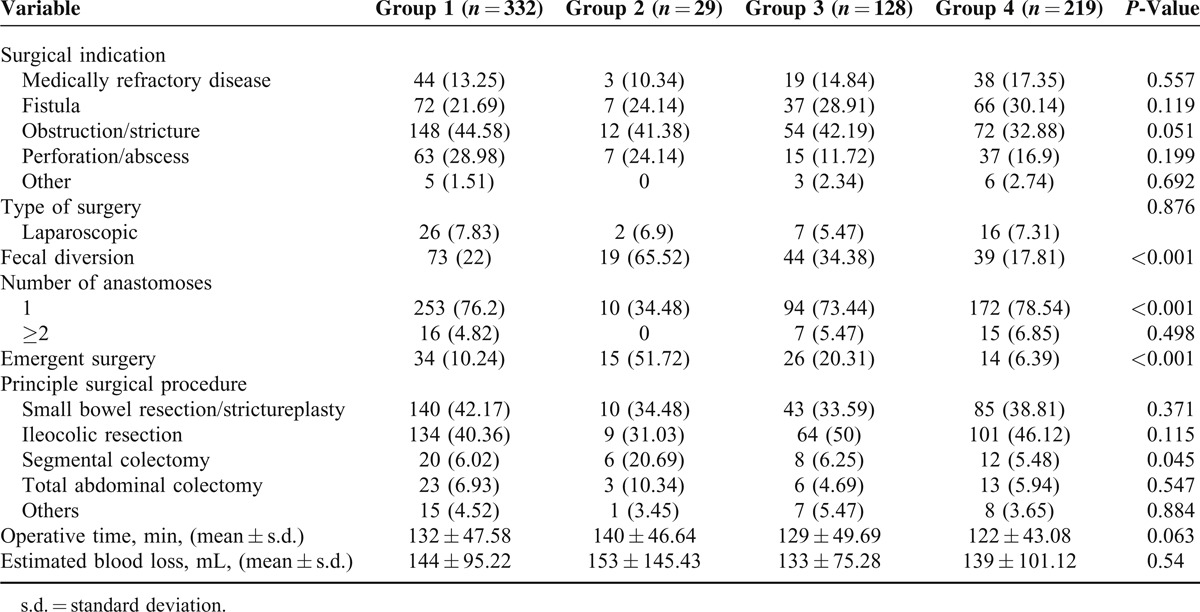
Operative Details of Abdominal Surgeries in Crohn's Disease Patients

### Postoperative Infectious Complications

**FIGURE 3:**
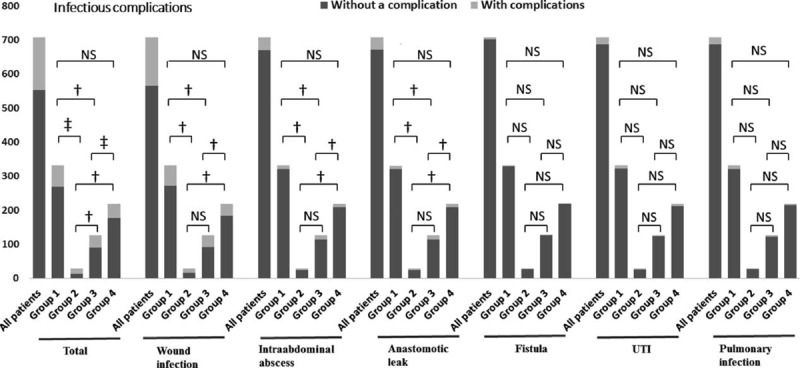
Postoperative infectious complications among groups. ^†^*P* < 0.05, ^‡^*P* < 0.001, and NS means not significant. UTI, urinary tract infection.

All 30-day postoperative infectious complications are shown in Table 3. There was a significant difference in the number of patients developing postoperative infectious complications among groups (Figure 3). There were significant differences among groups in wound infection, intra-abdominal abscess, and anastomotic leak. Higher frequencies of total infectious complications, wound infection, intra-abdominal abscess, and anastomotic leak were observed in group 2 and group 3. However, lower incidences of total infectious complications or any each specific infectious complication were found in group 4.

**TABLE 3:**
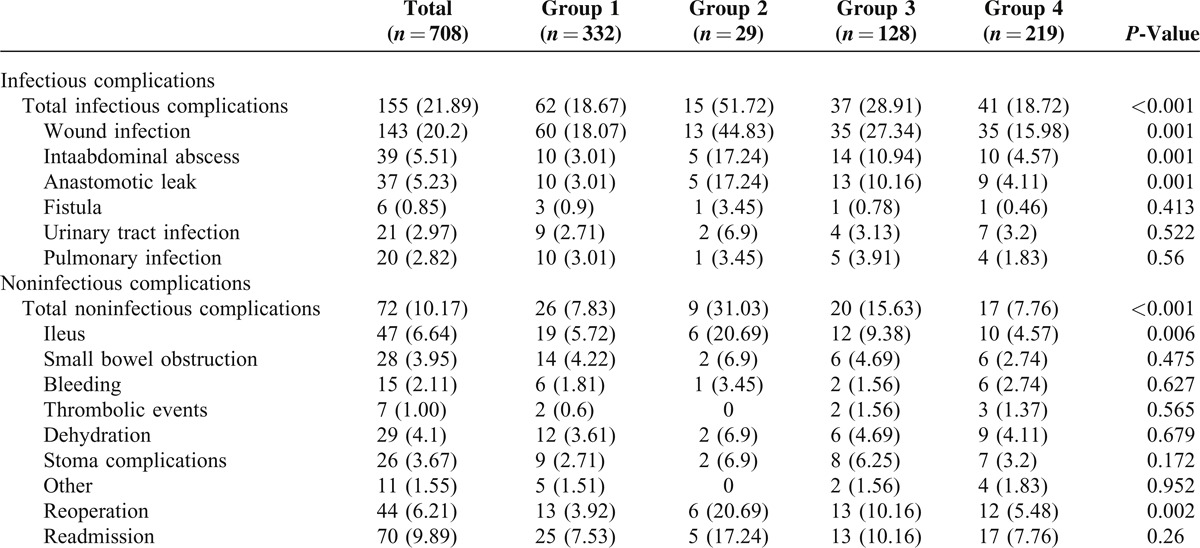
Postoperative Complications

### Noninfectious Complications

Postoperative noninfectious complications occurring within 30 days are presented in Table 3.

**FIGURE 4:**
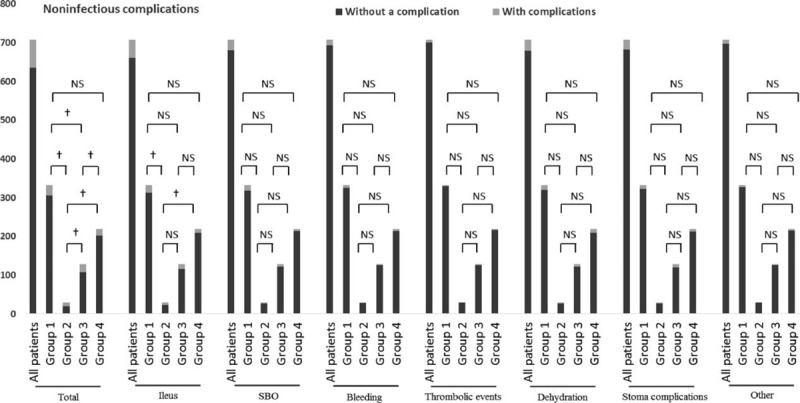
Postoperative noninfectious complications among groups. ^†^*P* < 0.05, ^‡^*P* < 0.001, and NS means not significant. SBO, small bowel obstruction.

There was a significant difference in respect to total noninfectious complications among groups (Figure 4). The group 2 and group 3 had higher frequencies of total noninfectious complications compared with group 1. However, no significant difference was found between group 1 and group 4 in respect to total noninfectious complications or any each specific infectious complication.

### Postoperative Reoperation and Readmission

**FIGURE 5:**
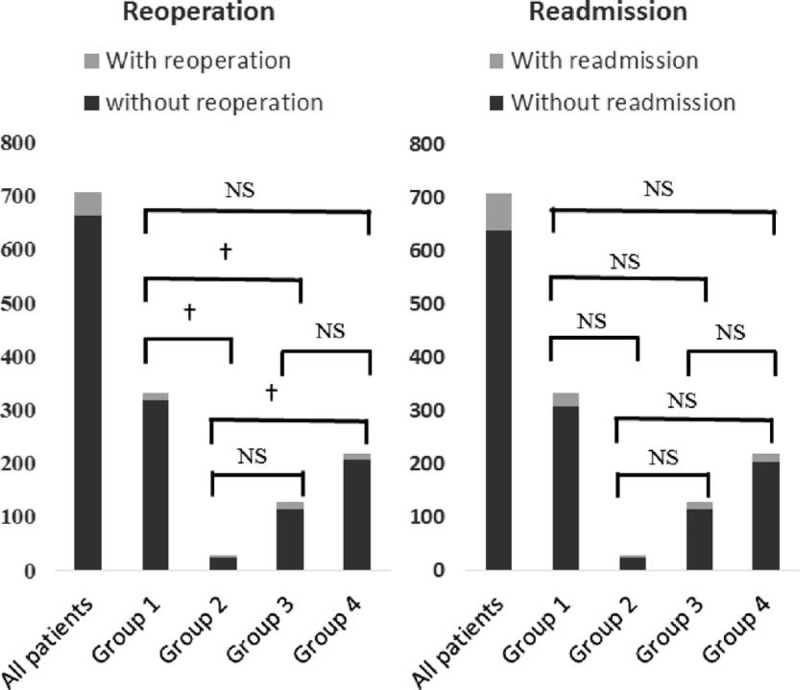
Reoperation and readmission among groups. ^†^*P* < 0.05, NS means not significant.

The overall reoperation rate is 6.21% (44/708). The rate of reoperation in group 1, group 2, group 3, and group 4 is 3.92% (13/332), 20.69% (6/29), 10.16% (13/128), 5.48% (12/219), respectively. There was a statistically significant difference among groups for reoperation (Figure 5). In our series, the overall readmission rate is 9.89% (70/708). The rate of readmission in group 1, group 2, group 3, and group 4 is 7.53% (25/332), 17.24% (5/29), 10.16% (13/128), 7.76% (17/219), respectively. There was no statistically significant difference among groups for readmission (Figure 5).

### Independent Risk Factors of Postoperative Complications

To identify the independent factor for postoperative complications. Multivariate logistic regression analysis model was used, and the results were shown in Table 4. The multivariate logistic regression analysis including risk factors such as preoperative immunosuppressive agent use, preoperative EEN, emergent surgery, CD-related surgical history, perforation/abscess surgical indication, longer operative time, and older age at surgery was performed. Our data indicated preoperative immunosuppressive agent use, emergent surgery, perforation/abscess surgical indication, and older age at surgery were found to be independent predictors for postoperative infectious complications, while preoperative EEN demonstrated to be a risk-reducing factor for infectious complications after surgery. In addition, in multivariate analysis, preoperative immunosuppressive agent use, perforation/abscess surgical indication, and longer operative time was independently associated with postoperative noninfectious complications, while preoperative EEN was found to be a protective factor for noninfectious complications after surgery.

**TABLE 4:**
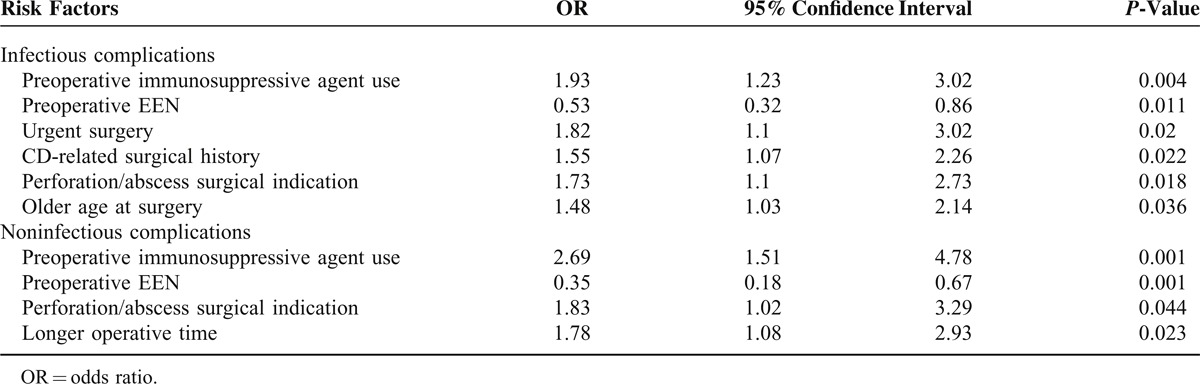
Multivariate Analysis of Factors Associated With Postoperative Complications

## DISCUSSION

A growing number of reports indicate the relative high postoperative morbidity and frequent infectious complications in patients with CD.^29–31^ Currently, immunosuppressive medications are often used in combination with one another for the preoperative treatment of CD.^32^ However, the safety of these immunosuppressive agents in perioperative setting is unclear. Accumulated data indicated that preoperative therapy with immunosuppressive agents increased the incidence of postoperative complications, particularly infectious and anastomotic leak.^11,33^ Therefore, preoperative drug window is needed for patients exposed to immunosuppressive agents with the purpose of drug wash out.

Our study found that, compared with those in other groups, patients without preoperative drug-free interval had increased incidences of both infectious and noninfectious complications, and higher risk of stoma creation. Preoperative EEN therapy provided longer drug-free interval, reduced the requirement of urgent surgical intervention, and decreased the rate of fecal diversion. In addition, postoperative complications in patients receiving EEN regimen were significantly decreased. We also found a higher rate of reoperation in patients without preoperative immunosuppresants-free interval although no significant difference was found between those with and without EEN regimen during the preoperative drug-free interval.

To date, few studies have evaluated the impact of preoperative drug-free interval on perioperative outcomes in patients with CD. The 1-month wash out period for azathioprine was reported based on the pharmacokinetics for the metabolite of the agent.^33–35^ For infliximab, 8-week wash out period was recommended according to pharmacokinetics and the treatment profile of the agent.^33,36^ In a meta-analysis, the authors concluded that patients with CD on preoperative immunosuppressive agents are at higher risk for complications, and a preoperative drug-free interval was suggested to reduce the risk of infections.^11^ In this study, we found preoperative immunosuppressants-free interval played an important role in decreased complications after surgery, and interestingly, we found the EEN regimen was associated with better outcome comparing with those without EEN during the drug-free interval.

Preoperative optimization of CD, when possible, may decrease postoperative complications, and avoid formation of stomas for fecal diversion.^37^ It is also reported that enhancing nutritional status and streamlining immunomodulator therapy before surgery may improve postoperative outcomes.^26^ EEN is the provision of 100% of a person's nutritional requirements which is effective in inducing remission in adults with active CD and proposed as an alternative to steroids therapy.^19^ Beside induction of remission, CD treatment is associated with remission maintenance as well as health-related quality of life.^24,38^ However, few studies were performed to evaluate the potential association between EEN and perioperative outcome in patients following immunosuppressive therapy although there are several studies focused on the association between total parenteral nutrition (TPN) and postoperative complications in CD.^39–41^ we found patients receiving adding EEN to preoperative drug-free interval regimen had a longer drug window. We may thus speculated that the longer preoperative immunosuppressants-free interval may contribute to the decreased incidence of postoperative complications. However, it was recently reported that increasing the washout period for biologicals to 12 weeks did not modify the frequency of postoperative surgical site infection.^42^ We tentatively put forward that EEN itself may play an important role in the decreased postoperative complications through inducing disease remission, attenuating the inflammatory response, and improving the nutrition status in patients with CD. Indeed, preoperative nutrition treatment was found to be associated with the reduced postoperative intra-abdominal septic complications.^21^ Based on our results, in clinical practice, for patients receiving immunosuppressive agents, preoperative drug-free interval should be recommended. If possible, patients can be treated with EEN regimen which is associated with longer drug window, decreased incidence of perioperative adverse events such as stoma creation and postoperative complications.

It should be noted that this study has inherent limitations. It is possible that those patients without preoperative EEN regimen are sicker patients who are intolerant to EEN therapy and are more likely to have postoperative complications. However, we think this is less likely considering the cohort study nature, and no difference was observed in respect to disease characters or operative information among groups. Second, <10% of our patients had a laparoscopic procedure, which may result in bias. This is probably a consequence of limited experience of laparoscopic procedure in patients with CD in China. Third, as the retrospective nature of the study, the abstraction of data especially medical records, may be subject to bias. In addition, because of its retrospective nature and no sample size calculation has been done, this long-term operated study may result in a potential selection bias. However, the data were extracted from the prospectively maintained IBD database, in which all variables were prospectively collected. Finally, there is a difference in immunosuppressive agents use for patients between China and other countries. None of our patients has been treated with budesonide or 6-mercaptopurine. Our results should be interpreted with caution when they are extrapolated to other patients.

In conclusion, despite its preliminary character, this study can clearly indicate that preoperative immunnosuppressants-free interval is required for Crohn's patients at increased risk for postoperative complications associated with the use of immunosuppressive agents. We found that preoperative optimization of CD following immunosuppressive therapy by EEN prolongs the immunosuppressants-free interval, reduces the risk of urgent surgery and reoperation, and most importantly, decreases complications after abdominal surgery. Our data offer a strategy to extend the preoperative immunosuppressants-free interval and thereby might be of benefit for a patient population at increased risk for postoperative complications associated with the use of immunosuppressive agents.
